# Effects of Size and Porosity on the Hydrophobicity of Hierarchical Nanoparticles

**DOI:** 10.1021/acs.nanolett.5c00058

**Published:** 2025-02-17

**Authors:** Yuriy G. Bushuev

**Affiliations:** Institute of Chemistry, University of Silesia in Katowice, 40-006 Katowice, Poland

**Keywords:** Hierarchical nanoporous materials, hydrophobic nanoparticles, Menger sponges, water intrusion/extrusion, structure of water

## Abstract

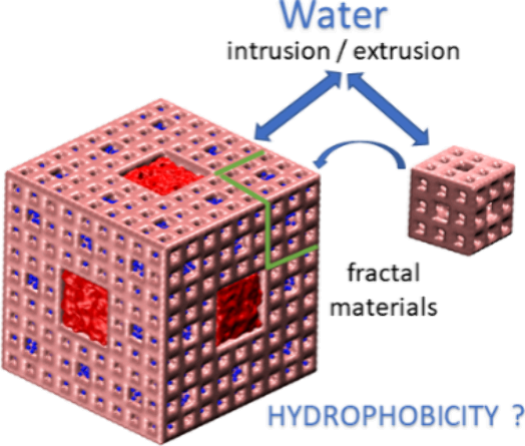

Hierarchical nanoporous particles combine properties of microporous and mesoporous materials that are widely exploited for energy storage and conversion, separation of gases and liquids, water purification and desalination, fabrication of nanodevices, etc. Hierarchical meso/microporous level-2 and level-3 Menger sponge particles immersed in water were investigated using computer simulation methods to demonstrate a synergetic effect of additional porosity on the wettability of materials. The Menger sponge is an object with a fractal dimension. At each level, the particles are composed of the same structural blocks. The hydrophobicity of the blocks was shown to depend on their size and position in the nanoparticles. The additional porosity decreases the hydrophobicity of the particles due to the partial breaking of hydrogen bonds between water molecules in the pores. This effect can be used to tune and modify the hydrophobicity and wettability of bulky porous materials, nanoparticles, and nanostructured surfaces.

Heterogeneous hydrophobic systems are composed of porous solids and water.^1,2^ The water wets hydrophobic micropores (a pore with an aperture smaller than 20 Å) under an elevated pressure. Mechanical work *P*_*int*_Δ*V*, where *P*_*int*_ is the intrusion pressure and Δ*V* is the volume of pores, must be produced to fill the pores with water. Depending on the system, the mechanical energy is totally or partially released when the pressure drops, and extrusion occurs. These systems are molecular springs or shock absorbers, respectively. They are called bumpers if the water stays in the pores after pressure release. The systems are used for energy storage and conversion,^3−7^ separating gases and liquids,^8^ water purification and desalination,^9,10^ and fabrication of nanodevices^11,12^ and in high-performance liquid chromatography.^13^

Many strategies are exploited^14^ to regulate surface wettability, which plays a significant role in determining the chemical and physical properties of materials. Chemical modification of surfaces by grafting with hydrophobic compounds^15^ results in significant improvements in the energetic performance of mesoporous materials,^16,17^ heat transfer, and reduced resistance to fluid flow in nanochannels. Due to control of surface morphology, superhydrophobic materials were produced.^18,19^ Regulation of the hydrophobicity of biological nanochannels is valuable for engineering biosensors and protein sequencing.^20−23^ The gating mechanism in biological systems depends on the wettability of hydrophobic pores.^24,25^ Chemically modified hydrophilic and hydrophobic nanoparticles are used for drug delivery and diagnosis and treatment of cancer.^26^

Thus, new methods for regulating the hydrophobicity of materials can help solve many scientific and technological challenges. It was demonstrated^27,28^ that the hydrophobicity of microporous materials depends on the topology of the porous system. The secondary porosity decreases the hydrophobicity of microporous materials, which can be tuned without any chemical modification of a material.

Microporous materials, such as metal–organic (MOFs),^29,30^ mesoporous silica,^16,31^ and zeolites,^32^ have various chemical compositions, where each element specifically interacts with water. Pure silica zeolites (PSZs) are hydrophobic crystalline solids composed of only silicon and oxygen atoms. Thus, they are more suitable for experimental investigations and computer simulations. However, the varieties of PSZ topologies, the sizes, shapes of pores, and chemical degradation of the materials after interaction with high pressure water hinder systematization and interpretation of the experimental results.

An attractive class of systems is hierarchical porous materials containing macro-, meso-, and micropores.^33−36^ The combination of various porosities in one material opens new avenues for their wide practical application. There is scarce information about the behavior of hierarchical porous hydrophobic zeolites in water and aqueous solutions. Some of them have been studied to increase the energetic performance of zeolitic systems.^37,38^ The results of experimental studies highly depend on the material synthesis procedure. Controlling the size and topology of additional irregular artificial pores in crystals is a challenge. Another problem regards the cyclization of water intrusion/extrusion processes. As a result of material degradation and cracking of the crystals, the properties of PSZ change. Broken −Si–O–Si– chains of bonds are terminated by dangling hydrophilic OH groups, which decrease the hydrophobicity of crystals.^39^

Computer simulations allow us to work with simplified systems, especially those designed to highlight the role of selected material characteristics. The Menger sponge,^40,41^ an object with a fractal dimension of 2.73, was chosen to answer what happens at an atomistic level when water penetrates hierarchical hydrophobic particles. A method for the synthesis of fractal silica materials was proposed.^42^ Aerogels are also fractal-sized materials, but because of their fragility, it is challenging to apply a high pressure to study water intrusion. Computer simulations of nanosized particles with a hierarchical porosity immersed in water are challenging. Thus, the simplified models are under investigation.

Classical molecular dynamics was exploited to simulate the intrusion and extrusion of water for the particles immersed in water. The Supporting Information (SI) presents the methodology of computer simulations in detail. The fcc crystalline structure of gold was used to model two levels, 2 and 3 cubic nanoporous particles, of the Menger sponge. Designated as the small particle (SP) and large particle (LP), respectively, these are presented in Figures 1a and S1. Both particles have a 3D system of channels with square cross sections and cubic cages at channel junctions. The LP consists of 20 SPs, which are elementary blocks of the LP. In turn, any SP consists of 20 level-1 Menger particles. The two types of micropores with openings of about 8 (the narrow pore) and 12 Å (the medium-sized pore) are in any SP. The LP has three intercrossed mesopores that form a six-way cross in the middle (Figure 1e). Each mesopore is a channel with square cross sections of about 36 Å aperture and more than 100 Å length (Figure S1). Thus, the LP is a hierarchical (mesoporous + microporous) particle. A priori, it is reasonable to expect the same hydrophobicity for all SPs independently of their position in the LP because the width, shape of pores, and interaction of pore walls with water are the same. A specially designed force field presented in the SI provided the hydrophobicity of the particles. This force field was exploited for simulations of nanotubes immersed in water.^43^ It was demonstrated^27^ that, for PSZ, calculated intrusion-extrusion isotherms are close to experimental ones if the SPC/Fw water model^44^ is used. The dependence of intrusion-extrusion isotherms on interactions of water with hydrophobic pores was demonstrated previously.^45^

**Figure 1:**
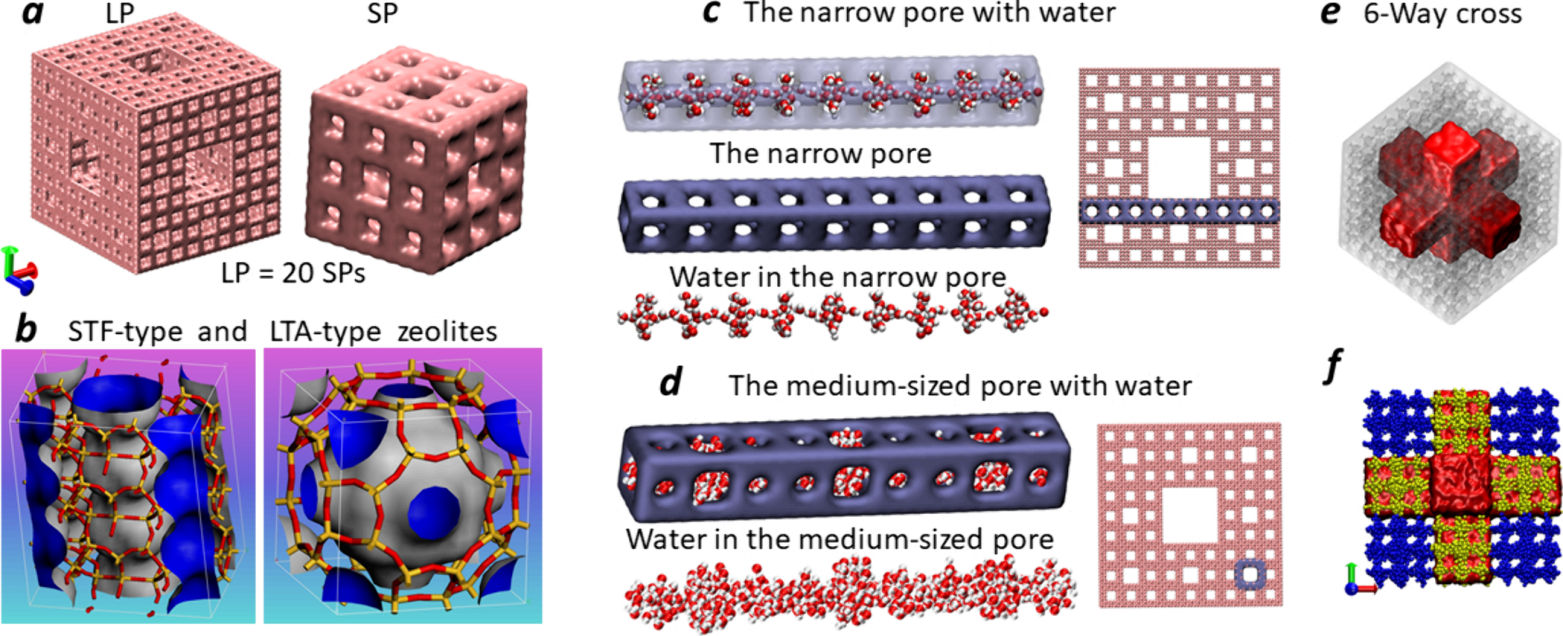
(a) Menger sponge nanoparticles level-3 (LP) and level-2 (SP) on an arbitrary scale. LP consists of 20 SPs. (b) Structure of STF- and LTA-type pure silica zeolites. The framework is formed by Si (yellow) and O (red) atoms. The external (gray) and internal (blue) surfaces show the shape of pores. (c) The longest narrow pore in the LP. (d) The longest medium-sized pore in the LP. (e) The 6-way cross is formed by intersecting mesopores (red) in the LP. (f) Corner (blue) and adjacent (yellow) cubes in the LP.

The regular structure of the particles and simplicity of interactions are advantages of the models. Meanwhile, they bear the characteristic features of zeolites. Figure 1b demonstrates the 1D channels in the STF-type PSZ. The channel looks like a sequence of beads on a string with a maximum diameter of an includible sphere of 7.6 Å. The 3D system of connected cages with a diameter of 11 Å is observed for LTA-type PSZ. Clusters of water molecules formed in the narrow and medium-sized pores of the LP under high pressure are presented in Figure 1c,d. They adopt the shape of pores and are similar to those in zeolites.^12,46,45^

The degree of wettability is determined by the contact angle between the liquid surface and the surface of the solid. However, in micropores, water cannot form surfaces, such as the meniscus. Individual intermolecular interactions and physical, geometric, and topological characteristics of pores determine the hydrophobicity of the materials.^27,28^ For example, PSZ with cages (LTA and others) are less hydrophobic than zeolites with a 3D system of channels. PZS with 1D channels (STF and others) are the most hydrophobic.^28^ The role of mesoporosity in the regulation of hydrophobicity of microporous materials is unknown. In the present work, wet and dry nanoparticles were investigated to shed light on the issue.

The calculated intrusion/extrusion isotherms for large and small particles immersed in water (Figure S2) are presented in Figure 2a as fractional loadings versus applied pressure. For this purpose, the number of water molecules in the particles was divided into the value calculated at 200 MPa. Water spontaneously permeates the mesopores at all pressures. These molecules were not taken into account when calculating the loadings of the LP. Thus, water penetrates only narrow and medium-sized pores under elevated hydrostatic pressure. Both systems demonstrate shock absorber behavior, with extrusion pressure less than an intrusion. Considering the same particle topology, geometrical characteristics, and water–particle interactions, one may expect that isotherms would be identical. However, there are three main differences in isotherms: (i) for the LP, intrusion occurs at a lower pressure, meaning that the SP is more hydrophobic than the LP; (ii) two-step extrusion is observed for the SP, corresponding to the sequential drying of narrow and medium-sized pores; (iii) for the SP, isotherms demonstrate more considerable intrusion-extrusion hysteresis. Thus, Figure 2a shows the effect of the particle size on the hydrophobicity of fractal nanoparticles.

**Figure 2:**
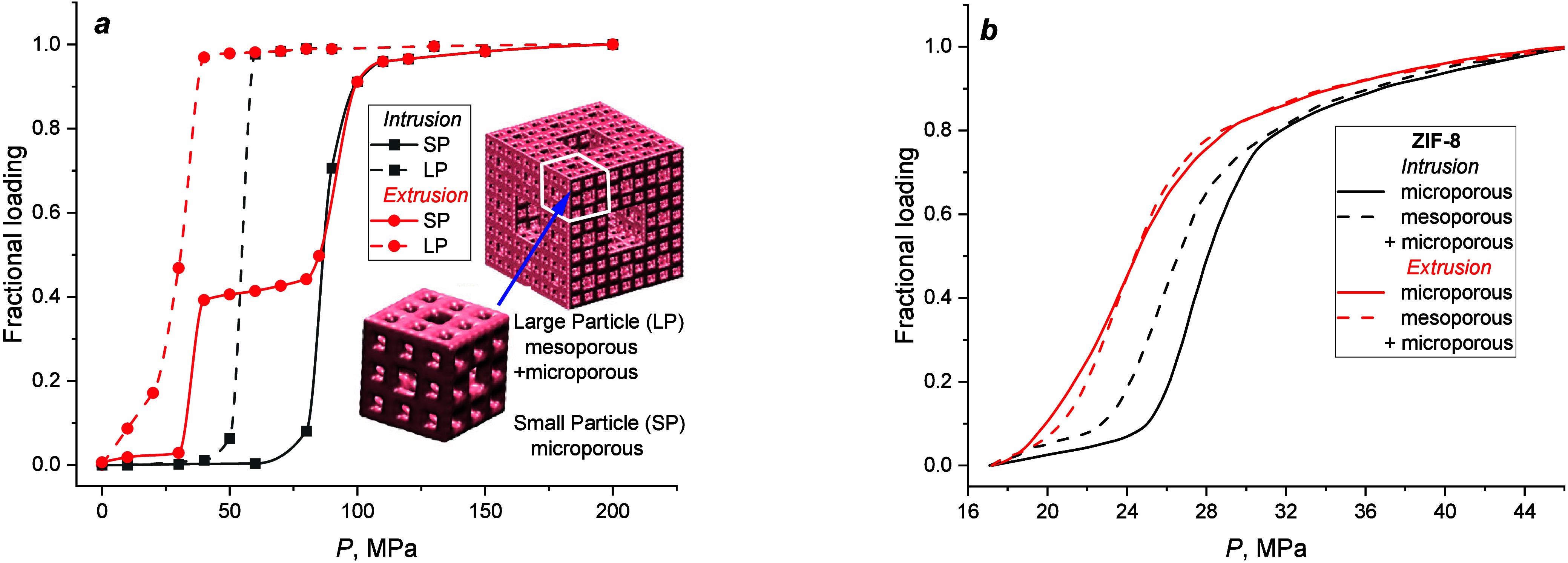
Water intrusion-extrusion isotherms: (a) for small and large Menger sponge particles and (b) for MOF ZIF-8 pristine microporous and hierarchical microsized crystals.

For comparison, experimental intrusion-extrusion isotherms^47^ for MOF ZIF-8 μm-sized crystals are presented in Figure 2b. The crystals with hierarchical porosity have a mean size of 2000 Å and mesopores of about 100 Å diameter, which, as in the case of mesopores of LP, are spontaneously filled with water at ambient pressure. Despite different chemical compositions, the geometry, the shape of pores, and the sizes of particles, there are some similarities in the behavior of the systems. Intrusion pressures are lower for particles with a hierarchical porosity. However, the shift in pressure is smaller for micrometer-sized crystals than for nanoparticles. The extrusion isotherms for the LP and hierarchical ZIF-8 have one step and are approximately the same as those for particles with only micropores (medium-sized pores in the SP). The intrusion-extrusion hysteresis is smaller when mesopores are present in the materials. Thus, the results of computer simulations do not contradict experimental ones and highlight the role of additional mesoporosity at various size scales.

MD simulations are allowed to explain the behavior of these heterogeneous systems. Insofar as the isotherms depend on particle size, the kinetics of water filling in the corner and adjacent cubes were investigated. Figures 1f and S1 show the positions of the cubes in both particles. They consist of 8 corner and 12 adjacent cubes. These are Menger sponges of level 2 (for the LP) or level 1 (for the SP). Previous simulations of water intrusion in zeolite with ITT topology have demonstrated the avalanche mechanism of water penetration.^27^ Intrusion started from the channels closest to the surface and propagated into the core of the crystal. In the LP, any corner cube has three medium-sized pores open to bulk water, whereas an adjacent cube has only two. Thus, it is reasonable to expect that water first enters the corner cubes. However, this assumption is incorrect.

Figure 3a,b demonstrates the kinetics of the penetration of water into the pores of both particles at various pressures. For this purpose, the mean numbers of water molecules in one cube of each type were calculated. As a result, on average, an adjacent cube contains more water than a corner one during intrusion. Identical numbers of molecules are in the cubes at the final stage if the applied pressure is higher than the intrusion pressure. The curves show that water wets adjacent cubes more easily. They are less hydrophobic than corner cubes.

**Figure 3:**
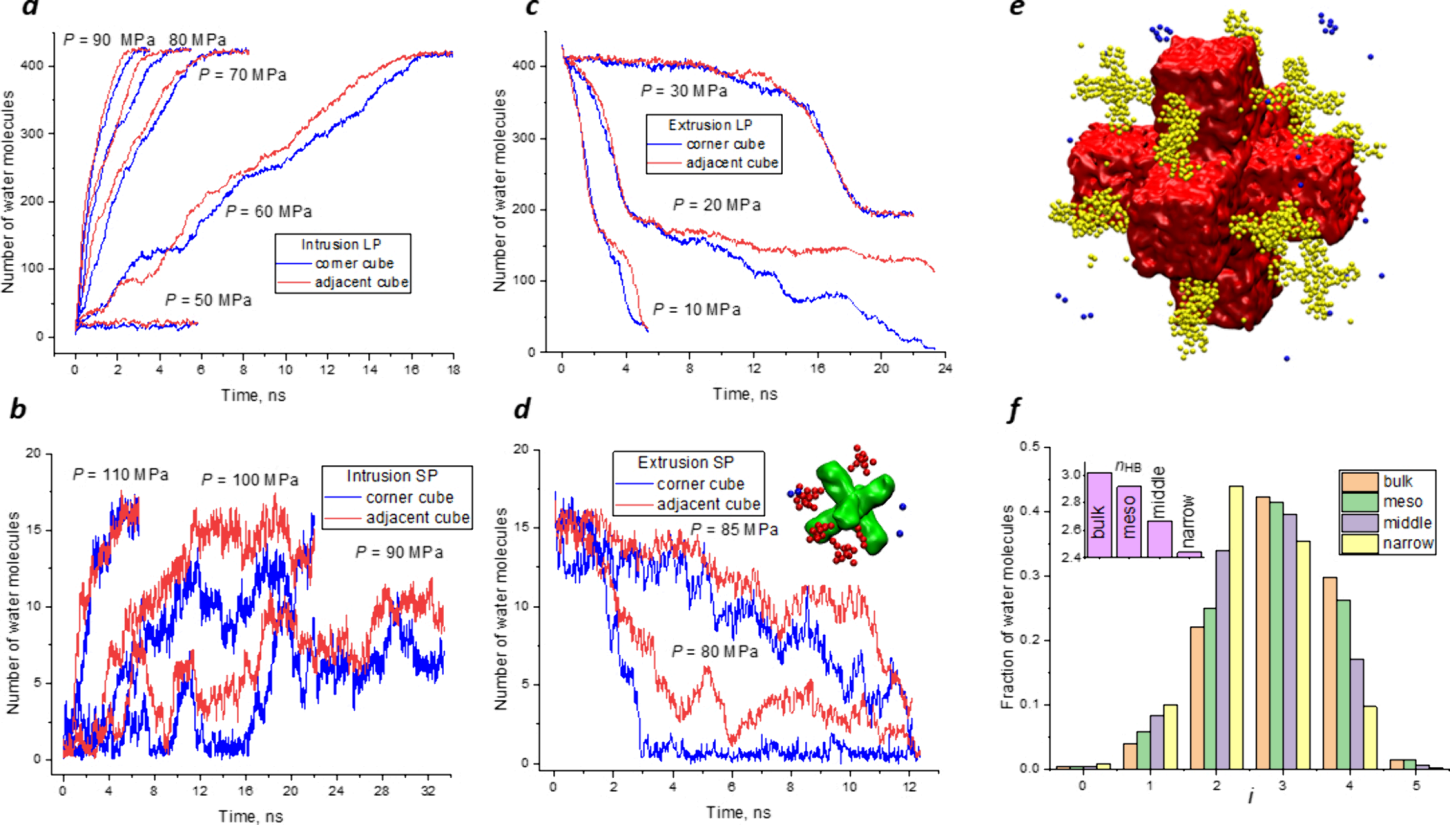
Time evolution of the number of water molecules intruding/extruding (a,b/c,d) into/from the corner and adjacent cubes of the large and small particles, respectively. (e) Water structures formed in the LP at *P* = 20 MPa and *t* > 16 ns. Water in mesopores is presented by red surfaces, water in adjacent cubes by yellow bolls, and water in corner cubes by blue balls. (f) Statistics of H bonds for bulk water and water in pores. Here, *i* is the number of hydrogen bonds in which a molecule participates and *n*_HB_ is the mean number of H bonds.

The plots demonstrating the kinetics of extrusion, presented in Figure 3c,d, support this conclusion. The two-step water extrusion from the LP is visible in Figure 3c, but in contrast to the SP, it occurs at a much lower pressure. Approximately half of the molecules left the particle in the first step. Initially, water exits through narrow pores. The narrow pores in the LP are less hydrophobic than in the SP. In the second step, the water leaves medium-sized pores. The corner cubes dry faster when the pressure is released. In the SP, the narrow pores in the corner cubes have no access to the medium-sized pores, and meanwhile, the medium-sized pores of the corner cubes in the LP have no access to the mesopores (Figures 1f and S1). Thus, additional porosity facilitates pore wetting and drying, thereby decreasing their hydrophobicity even if the wider pores spontaneously fill with water at atmospheric pressure, as in the case of mesopores in the LP.

It was shown^48−50^ that the observed intrusion-extrusion hysteresis is due to the metastability of water in pores. This state corresponds to the positions of points between the binodal and spinodal lines on the phase diagram of confined water. The transition from the filled to empty state of pores occurs in a narrow range of pressures. However, the kinetics is very slow near extrusion pressure, corresponding to half-loading. As a result, the curves presented in Figure 3c for 20 and 30 MPa may not reach equilibrium states during nanoseconds of simulations. However, the aim was to show the sequence of the pore drying. Longer simulation runs demand large amounts of computational resources. It can result only in a shift of positions of two points on the extrusion isotherm in Figure 2a, making the isotherm steeper. Still, this does not change the conclusion that medium-sized pores in the SP and the LP are dried at approximately the same pressure, in agreement with experimental data obtained for ZIF-8 (Figure 2b).

Small particles, the structural bricks of the LP, have different hydrophobicities depending on their positions. Figure 3e shows the formation of cross-like water clusters in adjacent cubes during extrusion. Water molecules in the medium-sized pore form a 4-way cross with access to water in mesopores through two openings. The other cross ends have access to bulk water. These water clusters are more stable and dry slower than other structures. Thus, wetting and drying of medium-sized pores depend on their access to other pores or bulk water. The most hydrophobic medium-sized pores are in the corner cubes. They do not have access to mesopores. The same effects are observed for the SP. Plots presented in Figure 3b,d show that narrow pores in adjacent cubes are wetted faster during intrusion and are dried slower during extrusion. Thus, the junction of pores with different apertures makes them less hydrophobic with respect to the pores, which are more accessible to bulk water.

The hydrophobicity of the SP depends on its position in the LP. The spontaneous collective reorientation of water molecules^43^ observed in long tubes, similar to those presented in Figure 1c,d, facilitates the fragmentation of water in pores and, after that, their drying. Short pores dry more quickly. The cube cages at the pore junctions, with thickenings similar to beads, stabilize water in micropores (Figures 1c,d and S3–S5). These are nucleation centers where water molecules are entrapped during intrusion/extrusion. This explains the two-step extrusion observed in Figure 2a for the SP. In the LP, water molecules can diffuse along a channel from one nucleation center to another without a large energetic barrier. The two-step extrusion is visible for the LP in the kinetic curves (Figure 1c), but it occurs at a lower pressure than for the SP. First, water leaves narrow pores but is entrapped at the nucleation centers of medium-sized pores (Figures S4 and S5). The SP has only one such center. These considerations explain the effect of pore length on hydrophobicity. Large nanoparticles are less hydrophobic than smaller ones.

The decrease in hydrophobicity observed for interconnecting pores with different widths can be explained by altering the water structure in the pores. For this purpose, the mean number of hydrogen bonds (*n*_HB_) and their statistics, the fractions of molecules forming *i* bonds with neighbors (*i* = 0–5), were calculated. In this work, the threshold criterion was applied. Two molecules are considered bonded if the energy of their interactions is less than −3.5 kcal/mol (*E*_HB_ < −14.64 kJ/mol).^12,43^ Thus, the strong- and medium-strength bonds are counted. The statistics of H-bonds are presented in Figure 3f. The mean number of bonds per molecule progressively decreases with the width of the pores, evidencing the alteration of the structure of water. However, in the central cube of the 6-way cross formed by mesopores (Figure 1e), the *n*_HB_ is the same as in bulk water. Meanwhile, *n*_HB_ is smaller for water in mesopores and micropores. The fractions of molecules with 0, 1, and 2 H bonds increase with the narrowing of the pores. Partial breaking of bonds due to nanoconfinement decreases fractions of molecules with 3, 4, and 5 bonds.

In the case of pore junctions, in order to permeate into a more narrow pore, water molecules must lose some hydrogen bonds that increase their energy. Thus, an energetic barrier for water penetration from a wider pore to a more narrow pore is lower than that of bulk water. The mechanical energy spent for system compression supports overcoming the barrier for micrometer-sized particles. However, in the case of nanoparticles, there are large fluctuations in pressure, which create fluctuations in the pore water loadings. This effect explains the sequence of wetting/drying of pores during water intrusion/extrusion into/from Menger sponges. Pores in adjacent cubes have more connections with pores where the hydrogen-bond network of water is partially broken than pores in corner cubes. Thus, the corner cubes are wetted slower than adjacent cubes during intrusion but are dried faster during extrusion.

Computer simulations of Menger sponges demonstrate that the hydrophobicity of porous nanomaterials depends not only on the geometry of pores, interactions of water with pore walls, and topology of the pore system but also on the size of nanoparticles, positions of structural blocks in hierarchical particles, and junctions of pores with different widths. The most hydrophobic elements are those in which the pores are more readily available to bulk water. Mesopores in hierarchical nanoparticles facilitate micropore wetting, making them less hydrophobic. The effect is explained by the increasing destruction of the hydrogen bond network of water with the narrowing of the pores. For micrometer-sized hierarchical particles, intrusion-extrusion isotherms must depend not only on pristine material samples but also on fabrication procedures of additional porosity. The hydrophobicity of nanoparticles or nanostructured surfaces can be tuned to a greater extent without chemical modifications of pristine materials. It can simplify the production of these materials, especially hybrid wettability surfaces.^15^ The new mechanism of hydrophobicity regulation can be exploited to design materials with targeted properties, especially nanoparticles for drug delivery, nanodevices, and nanostructured surfaces with improved mass and heat transfer, to manufacture a wide spectrum of new nanoporous materials with controlled and tuned wettability.

## Supporting Information Available

Details of MD simulation methods and additional figures (PDF)
DL_POLY input files for the small particle (ZIP)
DL_POLY input files for the large particle (ZIP)
