# Supplementary material for: Effects of Size and Porosity on the Hydrophobicity of Hierarchical Nanoparticles
Source: Nano Lett. 2025 Feb 17;25(8):3351–6. doi: 10.1021/acs.nanolett.5c00058 (PMC11869358; doi:10.1021/acs.nanolett.5c00058)
Supplement: Supplementary file 1 — nl5c00058_si_001.pdf [file nl5c00058_si_001.pdf]

SUPPORTING INFORMATION

Effects of Size and Porosity on the Hydrophobicity of Hierarchical Nanoparticles

Yuriy G. Bushuev

Institute of Chemistry, University of Silesia in Katowice, 40-006 Katowice, Poland

Method of computer simulations

The classical molecular dynamics method was exploited to simulate Menger sponges level 2 (SP) and level 3 (LP) immersed in water. Models of porous materials were produced from the face-centered cubic (fcc) crystal structure of gold. They are presented in Figure S1.

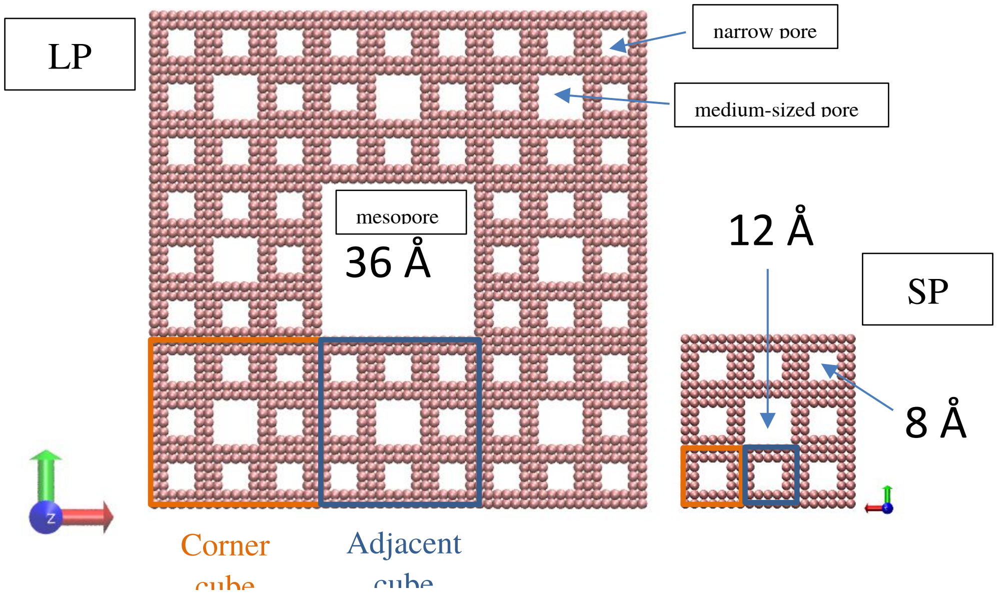

Figure S1. Structures of large (LP) and small (SP) particles and size of pore openings. Distances between centers of opposite atoms were measured.

A cubic simulation cell, presented in Figure S2, contained 1600 Au atoms and 1000 water molecules. 28928 Au atoms and 50000 water molecules were used to simulate the LP immersed in water. The atomic fractional coordinates of the porous particles were fixed during simulations carried out in an NPT ensemble at 300 K and various pressures ($0 \leq P \leq 200$ MPa). Due to a specifically chosen force field, the compressibility of the system was close to the compressibility of water. The average sizes of the pore openings are presented in Figure S1.

The force field providing the hydrophobicity of the particles was applied. The parameters of Lennard-Jones interactions between the oxygens of water and Au atoms are the same as between oxygens of water: $\varepsilon = 0.1554$ kcal/mol and $\sigma = 3.1655$ Å. The positions of Au were fixed. Thus, their interactions were neglected. The SPC/Fw flexible water model[1] was adopted for simulations with a recommended spherical cutoff radius of 9 Å. This force field was previously applied to simulations of nanotubes immersed in water.[2] The design of the model and the method of simulations were identical to the ones that were exploited early.[3] The Supporting Information is available free of charge at https://pubs.acs.org/doi/10.1021/acs.nanolett.1c02140. It contains results of simulations of water intrusion/extrusion in/from pure silica zeolites for various water models, flexibility of frameworks, and parameters of water – wall of pores interactions. Experimental support for our results was demonstrated.[3,4]

The DL_POLY version 5.1.0 code[5] was exploited to perform MD simulations. The smooth particle mesh Ewald method was used to calculate the long-range part of the electrostatic interactions. Equations of motion were numerically integrated using the velocity Verlet method with a time step of 1 fs. The duration of the runs depended on the intrusion/extrusion kinetics, reaching 24 ns in some cases. Examples of DL_POLY files containing all information about the simulation method and force fields are presented in SI.

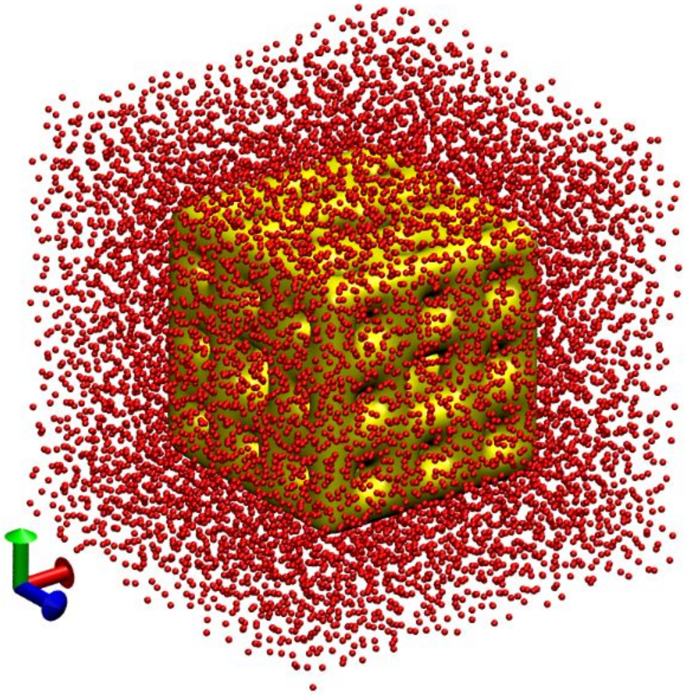

Figure S2. The small particle is immersed in water. Only oxygen atoms in the cubic simulation cell are presented for water. The SP is in the center of the cell.

Water intrusion/extrusion in/from the narrow pore of the large particle

The time evolutions of the system during intrusion and extrusion are presented in Figures S3 and S4, respectively. Only one long and narrow pore is highlighted in Figure S3. Intrusion starts at ca. 0.7 ns when water permeates in a fragment of the pore connecting the mesopore and the medium-sized pore. After 3 ns, the central fragment of the highlighted narrow pore, which has access to the mesopore, is filled with water. The pore is completely loaded after 5.2 ns. On average, the adjacent cubes fill up with water faster than the corner cubes.

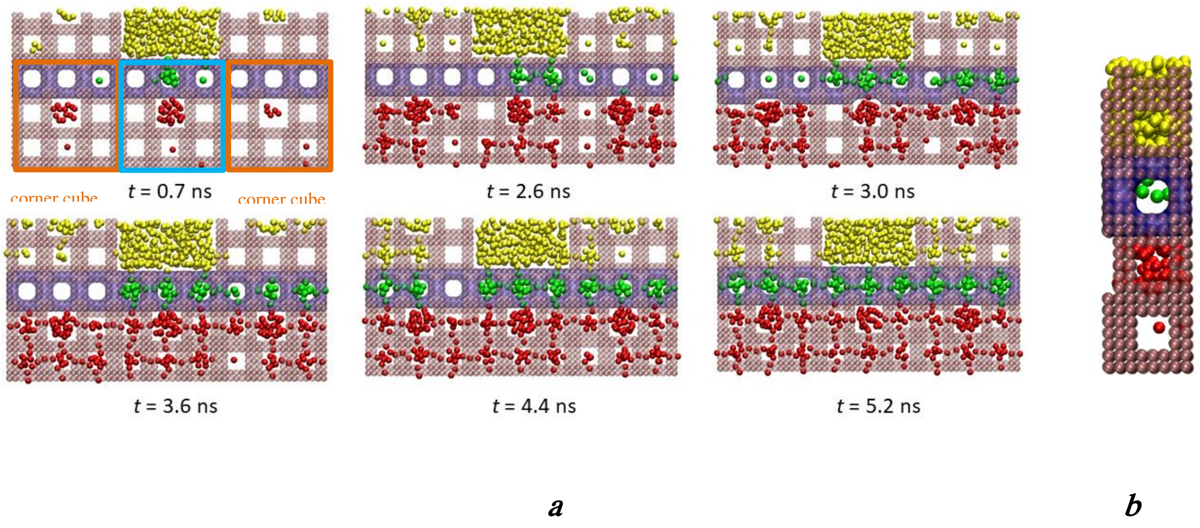

Figure S3. (***a***) Water intrusion in the LP at 70 MPa, *t* is the time. Only half of the particle is present. The micropores are empty, but the mesopores are filled with water at *t*=0.0 ns. (***b***) A side view of the particle fragment at *t*=0.6 ns is shown on a larger scale. The transparent blue surface highlights the narrow pore. Color scheme: The water molecules in the narrow pore are represented by green balls; yellow balls present water molecules above the narrow pore; red balls present water molecules below the narrow pore. Brown balls show the fragment of the LP.

Figure S3 demonstrates the role of mesoporosity in the wetting of micropores. The cubic centers of the micropores are the nucleation centers. They are filling in the early stage of water intrusion. The side view (Figure S3b) shows the water cluster (green balls) connecting the mesopore and the bulk water. At this moment, the medium-sized pore has been filled with water.

The stages of extrusion of water from two narrow pores at 20 MPa are presented in Figure S4. Here, two narrow pores are highlighted. The upper pore has fewer connections to the bulk water

and is more filled at *t*=1.1 and 1.8 ns. On average, the adjacent cube contains more water than the corner cubes. At 3 ns, the last stage of drying of narrow pores is presented. Water is in fragments of pores that connect to medium-sized pores or mesopores.

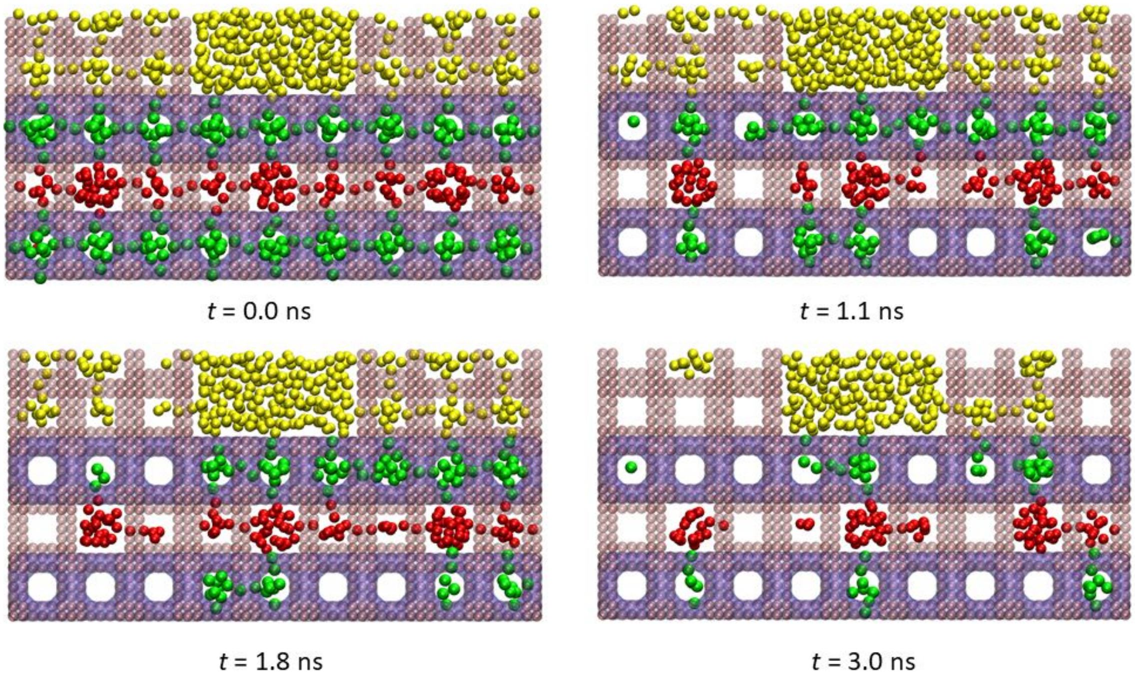

Figure S4. Extrusion of water from the LP at 20 MPa, *t* is the time. Only half of the particle is present. The transparent blue surfaces highlight narrow pores. Color scheme: Water molecules in the highlighted narrow pores are presented by green balls; yellow balls present water molecules above the upper narrow pore; water molecules between narrow pores are presented by red balls. Brown balls show a fragment of the LP.

Water extrusion from the medium-sized pores of the large particle

Figure S5 shows the evolution over time of the water clusters in medium-sized pores. At 2.4 ns, water has left narrow pores, but most medium-sized pores are filled with water. At 3.8 ns, most clusters that had a 6-way cross shape were transformed into 4-way crosses connecting mesopores with bulk water. They are mainly in the adjacent cubes. At 4.6 ns, the less stable clusters left the pores. Most of the corner cubes are dry.

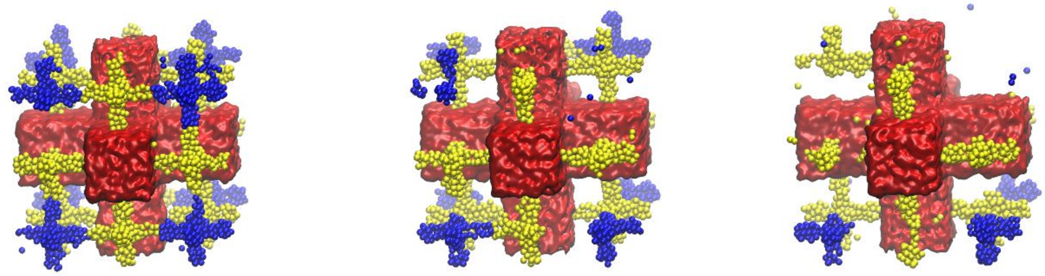

Figure S5. Extrusion of water from LP at 10 MPa after 2.4, 3.8, and 4.6 ns. Water in mesopores is presented by red surfaces, water in adjacent cubes by yellow balls, and water in corner cubes by blue balls.

References.

(1) Wu, Y.; Tepper, H. L.; Voth, G. A. Flexible Simple Point-Charge Water Model with Improved Liquid-State Properties. *J. Chem. Phys.* **2006**, *124*, 024503. https://doi.org/10.1063/1.2136877.

(2) Bushuev, Y. G.; Grosu, Y.; Chorążewski, M. Spontaneous Dipole Reorientation in Confined Water and Its Effect on Wetting/Dewetting of Hydrophobic Nanopores. *ACS Appl. Mater. Interfaces* **2024**, *16* (6), 7604–7616. https://doi.org/10.1021/acsami.3c17272.

(3) Bushuev, Y. G.; Grosu, Y.; Chorążewski, M. A.; Meloni, S. Subnanometer Topological Tuning of the Liquid Intrusion/Extrusion Characteristics of Hydrophobic Micropores. *Nano*

Lett. **2022**, *22* (6), 2164–2169. https://doi.org/10.1021/acs.nanolett.1c02140.

(4) Lowe, A. R.; Chorążewski, M. A.; Grosu, Y.; Bushuev, Y. G. Energetic Characteristics of Hydrophobic Porous Materials as Candidates for Manufacturing of Nanorockets˙. *J. Phys. Chem. Lett.* **2024**, *15*, 12112–12119. https://doi.org/10.1021/acs.jpclett.4c02581.

(5) Todorov, I. T.; Smith, W.; Trachenko, K.; Dove, M. T. DL_POLY_3: New Dimensions in Molecular Dynamics Simulations via Massive Parallelism. *J. Mater. Chem.* **2006**, *16* (20), 1911–1918. https://doi.org/10.1039/b517931a.
